# Assessing ischemic myocardial metabolism in vivo with hyperpolarized 13C: relating the metabolic perturbation to the area at risk

**DOI:** 10.1186/1532-429X-17-S1-O97

**Published:** 2015-02-03

**Authors:** Hikari Yoshihara, Jessica A Bastiaansen, Corinne Berthonneche, Arnaud Comment, Juerg Schwitter

**Affiliations:** 1 Dept. of Cardiology, Lausanne University Hospital (CHUV), Lausanne, Switzerland; 2 Dept. of Radiology, Lausanne University Hospital (CHUV), Lausanne, Switzerland; 3 Cardiovascular Assessment Facility, Lausanne University Hospital (CHUV), Lausanne, Switzerland; 4 Institute of Physics of Biological Systems, Swiss Federal Institute of Technology (EPFL), Lausanne, Switzerland

## Background

The high metabolic activity of the heart makes it particularly suited to the use of hyperpolarized (HP) 13C methods to non-invasively detect and characterize metabolic changes that occur during ischemia/reperfusion (I/R). Energy metabolism in ischemic rat hearts has been previously interrogated with HP 13C-labelled pyruvate ex vivo, and its hyperpolarized metabolites have been imaged in the ischemic pig heart in vivo. In both cases, a decrease in the conversion to labelled bicarbonate was observed vs. conversion to lactate, consistent with the expected decrease in pyruvate oxidation. Here, our aim was to establish this I/R model in rats and to correlate metabolic changes with the area at risk.

## Methods

The femoral arteries and a vein were catheterized in anaesthetized, intubated Wistar rats (n=16, 265.9 ± 3.1 g). After positioning the rat in the MR scanner (Varian, 9.4T) and shimming, a solution of HP [1-13C]pyruvate was infused. A series of 40 single pulse (300 flip angle) 13C MR spectra was then acquired (gated by pulse and respiration, TR ~3s) using a surface coil over the heart, to establish the baseline state. Myocardial ischemia was effected by occlusion with a snare installed around the left coronary artery, in place for 15 min (omitted in controls), followed by another HP infusion and spectral acquisition. To determine the size of area at risk, the heart was stained with Evans blue. Spectral peaks were quantified by fitting, and areas under curve (AUC) for metabolite signal time courses were calculated using the spectral signal amplitude and the time between gated acquisitions. Statistical significance was calculated by one-way ANOVA.

## Results

**Figure 1:**
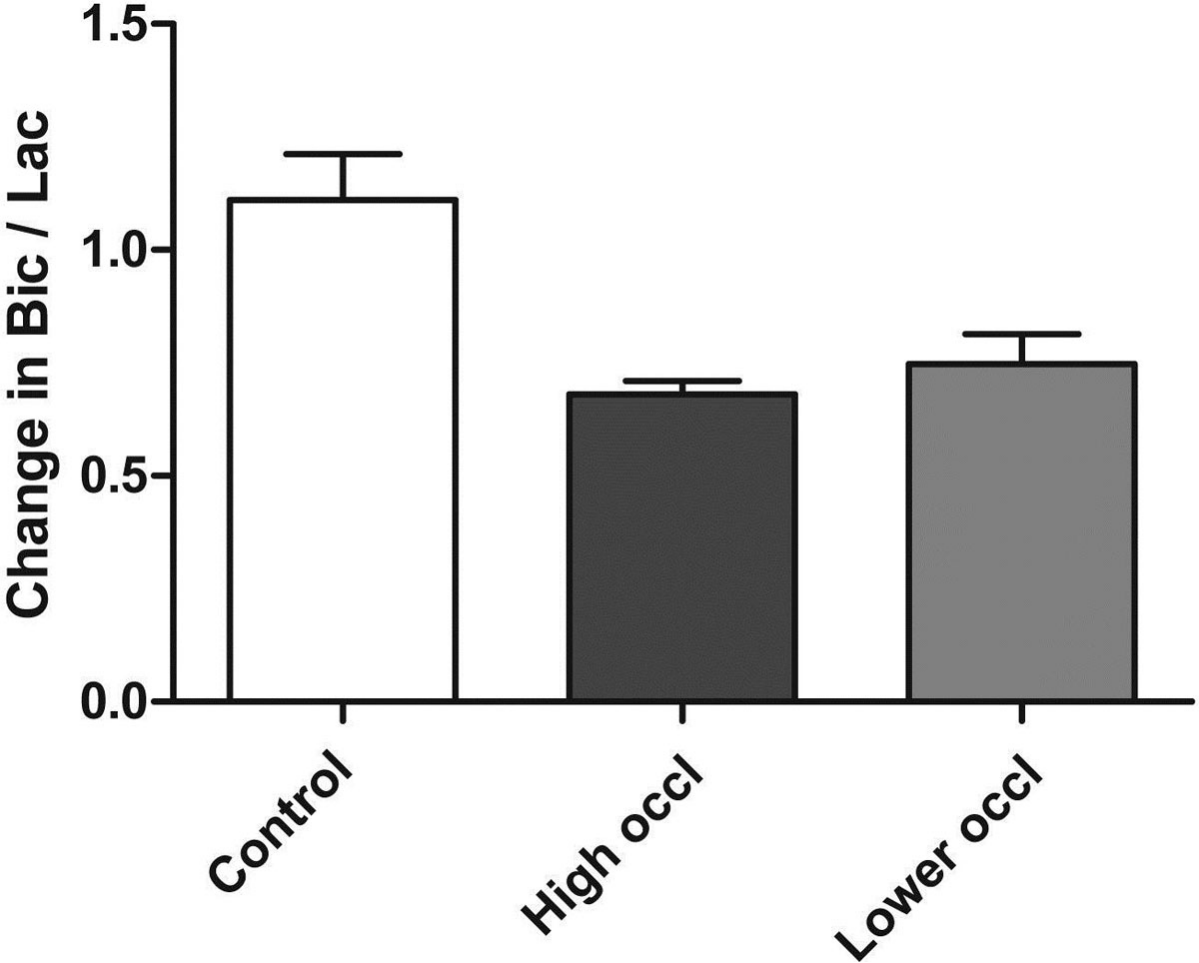
Change in hyperpolarized 13C-bicarbonate-to-[1-13C]lactate ratio following 15 min period of ischemia, compared to baseline. In control experiments, bic-to-lac ratio is near baseline level. After ischemia, the ratio is significantly lower. Occluding higher on LAD results in large area at risk, while occluding lower results in a more variable area at risk and variable metabolic change.

**Figure 2:**
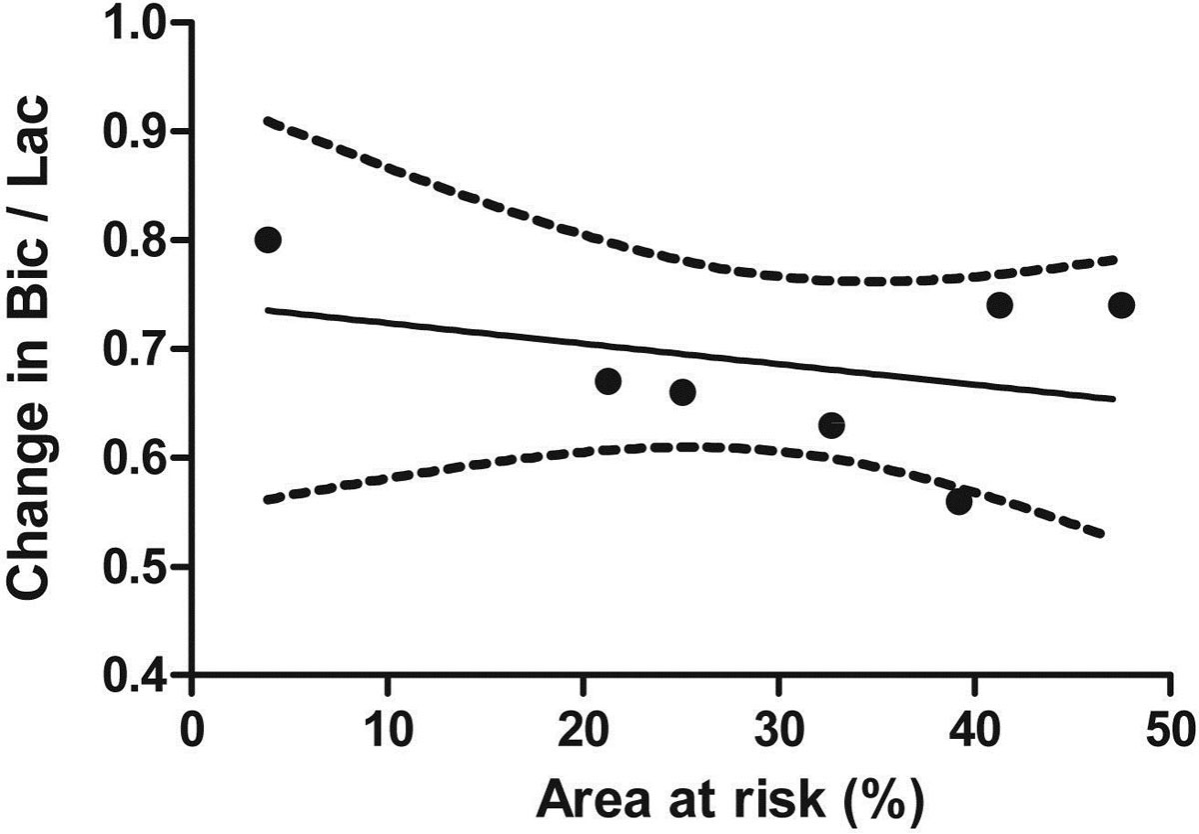
Change in hyperpolarized 13C-bicarbonate-to-[1-13C]lactate ratio following 15 min period of ischemia with occluding thread placed lower on LAD, compared to size of area at risk measured by Evans blue staining. Solid line shows linear regression with 95% confidence interval in dotted lines.

The HP metabolites [1-13C]lactate, [1-13C]alanine and 13C-bicarbonate were detected before and after myocardial ischemia. The 13C-bicarbonate-to-[1-13C]lactate (Bic-to-Lac) ratio was 0.68 ± 0.03 SEM the level of baseline (n=3), compared to 1.11 ± 0.10 in control experiments (n=5) (p=0.02), reflecting the shift from oxidative metabolism to anaerobic metabolism. (Figure 1) To reduce the mortality rate, another set of experiments was performed with the occluding thread placed more distally on the coronary artery; this resulted in a similar decrease in the Bic-to-Lac ratio, 0.75 ± 0.07 compared to baseline (n=8) (p=0.01 vs. control). The variability in the metabolic perturbation of the latter group was compared to the size of the area at risk, which ranged from 4.4 to 47.5% of the heart, (Figure 2) and shows a trend towards a greater metabolic change with a larger area at risk, but with significant variability.

## Conclusions

This study demonstrates the feasibility of using HP 13C MRS to detect metabolic changes in rat myocardial metabolism in vivo after a brief ischemic episode and suggests the possibility of spectroscopically estimating the extent of injury. It provides a platform to investigate future treatment strategies to reduce reperfusion injury.

## Funding

Work supported by the Swiss National Fund (grants #138146 & PPOOP1_133562).

